# Video-Based Feeding Demand Sensing and Offline Feeding Strategy Evaluation in Group-Housed Pigs: A Single-Pen Proof-of-Concept Study Using Oriented Object Detection

**DOI:** 10.3390/ani16142219

**Published:** 2026-07-17

**Authors:** Xinyuan He, Weijia Lin, Guoxing Chen, Yanhua Liu, Enli Lyu, Ziwei Li, Yizhi Luo, Zhixiong Zeng

**Affiliations:** 1 College of Engineering, South China Agricultural University, Guangzhou 510642, China; xin_yuan_he@163.com (X.H.); wekalin@163.com (W.L.); guoxing.chen@scau.edu.cn (G.C.); cynthial@scau.edu.cn (Y.L.); enlilv@scau.edu.cn (E.L.); lzw20090923@163.com (Z.L.); 2 Institute of Facility Agriculture, Guangdong Academy of Agricultural Sciences, Guangzhou 510640, China; luoyizhi@gdaas.cn

**Keywords:** precision livestock farming, group-housed pigs, intelligent feeding, feeding behavior recognition

## Abstract

Continuous sensing of group feeding demand is needed to support feeding decisions on large-scale pig farms. In dense group-housing environments, crowding, occlusion, and variation in posture around the trough can reduce the stability of visual feeding-status recognition. Feeding schedules also commonly rely on fixed periods or historical patterns, limiting their ability to assess whether daily feeding-behavior signals are aligned with group demand. Here, we propose a video-based workflow for sensing group feeding activity and conducting an offline replay evaluation of feeding-schedule allocation rules in group-housed pigs. Using synchronized feeding videos and feeder records, we developed a workflow that integrates rotated object detection, visual feeding intensity calculation, time-period-level construction of video-derived feeding demand, and feeding strategy evaluation. Rotated bounding-box detection achieved an mAP50-95 of 0.908 for feeding recognition, supporting its feasibility for feeding/non-feeding recognition in this dense-pen scene. Visual feeding intensity derived from model outputs was most strongly correlated with actual feed intake increment under a 20 min aggregation window with no time lag (r = 0.80). In the evaluation based on video-derived feeding demand, the video-perceived hybrid strategy reduced the peak time difference to 14.51 min, achieved a synchronization correlation of 0.611, and reduced the mismatch rate to 5.13%. These findings suggest that video-based behavioral signals can serve as a group-level proxy for feeding demand and can support offline evaluation of behavior-aware feeding schedules. However, the demand and strategy analyses were based on a single pen observed for 72 h, and validation across additional pens, batches, age stages, seasons, and closed-loop feeding conditions is required before practical deployment.

## Simple Summary

On large pig farms, feeding is often scheduled at fixed times or based on past experience, although pigs’ demand for feed can change during the day. As a result, feed may be delivered too early, too late, or at times when the group has low feeding demand. This study tested whether video recordings could be used to track real-time feeding demand in group-housed pigs. A computer-vision model was used to identify pigs that were feeding, and the number of feeding pigs was converted into a feeding-intensity signal. This signal was then compared with feeder records. The method detected feeding behavior reliably in crowded pens. The video-derived feeding intensity closely matched actual feed intake, with a correlation of 0.80 over 20 min time windows. When used in an offline replay evaluation, the best video-assisted allocation rule reduced the difference between the video-derived demand peak and the planned feeding peak to 14.51 min and lowered the mismatch rate to 5.13%. These results suggest that video-based monitoring can provide a behavioral feedback signal for designing feeding schedules. Because the demand and scheduling analyses were based on one pen observed for 72 h, further validation across pens, batches, seasons, and closed-loop feeding conditions is required before practical deployment.

## 1. Introduction

As large-scale pig production moves toward precision, automation, and smart management, feeding management is no longer confined to meeting the daily feed allowance. It is increasingly shifting toward continuous sensing of animal status and production processes to support management decisions [1,2]. Advances in sensors, video monitoring, automated feeding equipment, and data analytics have provided a technical basis for recording feed intake, adjusting nutrient supply, and monitoring production status [3,4,5,6]. Previous studies on precision feeding have shown that estimating nutrient requirements from real-time or near-real-time data can help optimize feeding management and may improve feed-use efficiency and resource utilization [7,8]. Fixed feeding schedules are simple to implement, but they cannot respond to short-term changes in group feeding activity caused by behavioral rhythm, feeder competition, management disturbance, or environmental variation. Under group-housing conditions, the same daily feed allocation may therefore be delivered at periods that do not match the current demand curve. A behavior-aware schedule has practical engineering value because it converts continuous animal-state sensing into period-level feedback for deciding when feed should be released under existing equipment and daily allocation constraints [9,10].

Feeding behavior in group-housed pigs is jointly influenced by age, feeding space, feeding strategy, nutrient supply, and individual differences. It therefore often exhibits clear diurnal variation and within-group heterogeneity [11,12]. Feeding behavior indices, feeding structure, and feeding rhythms have been used to explain variation in growth performance, welfare status, and feed efficiency in finishing pigs [13,14,15,16]. Electronic feeding systems and RFID-based devices have also improved automatic recording of feeding behavior [17,18]. However, these records mainly reflect feeding events or outcomes. They may not fully capture the immediate behavioral state of pigs near the trough. For intelligent feeding systems that require dynamic within-day adjustment of feed allocation, a continuous, low-intrusion group-behavior feedback signal that can be updated in real time is still needed.

Video monitoring offers non-contact sensing, continuous coverage, and rich scene information. It has therefore been increasingly used for feeding-behavior recognition, posture detection, and group-behavior analysis in pigs [19,20,21]. Two-dimensional cameras combined with deep learning models have shown potential for recognizing pig location, posture, drinking behavior, and health status [22,23,24,25]. However, visual recognition in farming environments still faces several challenges, including dense animal distribution, partial occlusion, illumination variation, changes in posture orientation, and ambiguous behavioral boundaries. More complex tasks, such as tail biting, contact behavior, risky behavior, and group video monitoring, place even higher demands on object localization, temporal representation, and cross-scene robustness [26,27,28,29].

Stable recognition of the number of pigs in a feeding state from video could form the basis of continuous indicators of group feeding intensity. Such indicators may offer a more timely representation of feeding demand than static plans or historical averages. In recent years, markerless pose estimation, individual identification, and object tracking have expanded the application scope of visual sensing in precision pig farming [30,31,32]. Oriented bounding boxes can better fit the long axis and boundaries of targets. They may therefore offer advantages for detecting objects in dense scenes with variable orientations [33,34,35]. However, their applicability to feeding-behavior recognition and feeding-demand representation in group-housed pigs remains insufficiently validated. Converting frame-level feeding-recognition results into engineering indicators aligned with actual feed intake remains a key issue. These indicators must also support feeding scheduling in intelligent feeding systems.

To address these issues, we developed a video-based framework for sensing group feeding activity and conducting an offline evaluation of feeding-schedule allocation rules in pig groups. The framework first casts individual pig localization and feeding/non-feeding state recognition as an object detection task. A visual model is then used to derive the frame-level count of feeding pigs. These outputs are aggregated within predefined time windows to calculate visual feeding intensity. The indicator is then temporally aligned with feed intake increments recorded by intelligent feeders to evaluate its ability to represent intraday variation in group feed intake. Finally, we summarized visual feeding intensity within each feeding period to obtain a period-level indicator of video-derived feeding demand. We then used this indicator in an offline replay analysis to compare how well different allocation rules matched feeding demand over time, while keeping the total daily feed amount and the maximum feed share per period fixed.

The main contributions of this study are threefold:

1. In dense group-feeding scenarios in pigs, we systematically compare horizontal bounding box (HBB) and oriented bounding box (OBB) detection formats for feeding/non-feeding state recognition, and evaluate several detection architectures. The results indicate an overall advantage of YOLO26-OBB in balancing detection accuracy and inference efficiency.
2. We aggregate frame-level feeding-recognition results into visual feeding intensity and evaluate its association with feed-intake increments using multi-scale correlation analysis and leave-one-day-out cross-validation.
3. We construct a period-level video-derived feeding-demand indicator and use it for offline feeding-schedule evaluation. Specifically, we compare the temporal match between candidate allocation rules and video-derived demand under fixed total daily feed allocation and per-period allocation constraints.

The remainder of this paper describes data collection and methodological design, visual recognition and feeding-intensity construction, and feeding-strategy evaluation. We then discuss the engineering significance of the proposed method in intelligent feeding systems, together with its limitations.

## 2. Materials and Methods

### 2.1. Data Collection and Image Annotation

#### 2.1.1. Data Collection

The data used in this study were collected from a group-housed pig feeding-monitoring scenario on a pig farm in Yunnan, China. The pen was 3.6 m long and 4.8 m wide, and housed 30 crossbred growing pigs. Videos were recorded using a Batianan camera (BTA-DS-400JA-AK, Guangzhou Zhuobei Intelligent Technology Co., Ltd., Guangzhou, China) installed above the side of the pen. Video recording lasted for 72 h, during which the pigs were 59–62 days old. The video resolution was 2560 × 1440 pixels, and the frame rate was 25 fps. Actual feed intake was recorded using an automatic feeder installed in the middle of the pen (IF3000, Jiaen Technology, Guangzhou, China). The recording interval was approximately 5 min. To ensure temporal consistency between visual behavior data and feed-intake data, video frames and feed-intake records were aligned using device timestamps. Visual feeding behavior and actual feed-intake increments were then summarized within fixed time windows.

#### 2.1.2. Image Annotation

To reduce redundancy caused by prolonged feeding or lying postures, frames were extracted from the 72 h videos at 1 s intervals. From these candidate frames, 1200 valid images were selected to cover different observation days, feeding and non-feeding periods, animal densities near the trough, postures, occlusion levels, and illumination conditions. Only pigs whose visible body boundaries and feeding status could be reliably determined were annotated; pigs outside the field of view, fully occluded, or insufficiently visible for reliable boundary or class assignment were excluded. In total, 30,923 pig instances were annotated, including 4545 feeding instances and 26,378 non-feeding instances. All targets were annotated as oriented bounding boxes using X-AnyLabeling v3.3.7 [CVHub, https://github.com/CVHub520/X-AnyLabeling (accessed on 13 January 2026)] and manually reviewed [36]. Feeding was defined as a visible pig whose head or mouth was engaged with the trough or feed area and whose posture was consistent with active feeding. Non-feeding included standing, walking, lying, or remaining near the trough without visible feeding engagement. Ambiguous cases were reviewed according to these criteria. The annotated dataset was divided into training, validation, and test sets at a ratio of 8:1:1.

### 2.2. Overall Research Framework

**Figure 1:**
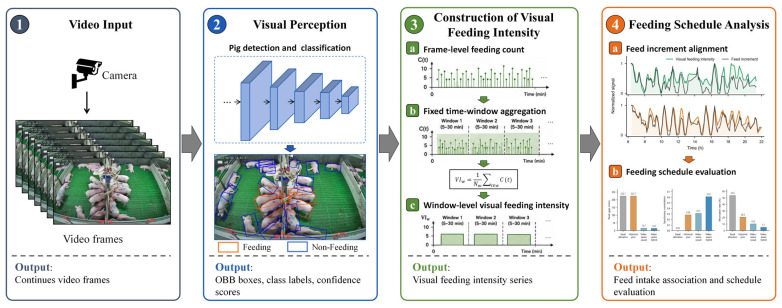
Research framework for feeding-demand sensing and scheduling optimization in group-housed pigs.

This study developed a visual analysis framework for sensing feeding demand and evaluating feeding strategies in pig groups, as shown in Figure 1. First, an object detection model was used to identify individual pigs and their feeding/non-feeding states in video frames, generating frame-level feeding counts. These counts were then aggregated into a visual feeding intensity sequence using fixed time windows. The sequence was temporally aligned with actual feed intake increments from the same period. This alignment was used to assess whether visual behavioral signals could represent intraday changes in group feed intake. Based on this analysis, visual feeding intensity was further summarized at the feeding-period scale to construct a video-based feeding-demand indicator. Under a constant total daily feed allocation, temporal matching was evaluated between planned feed allocation under different feeding strategies and video-based feeding demand.

### 2.3. Feeding Recognition Model and Training Settings

**Figure 2:**
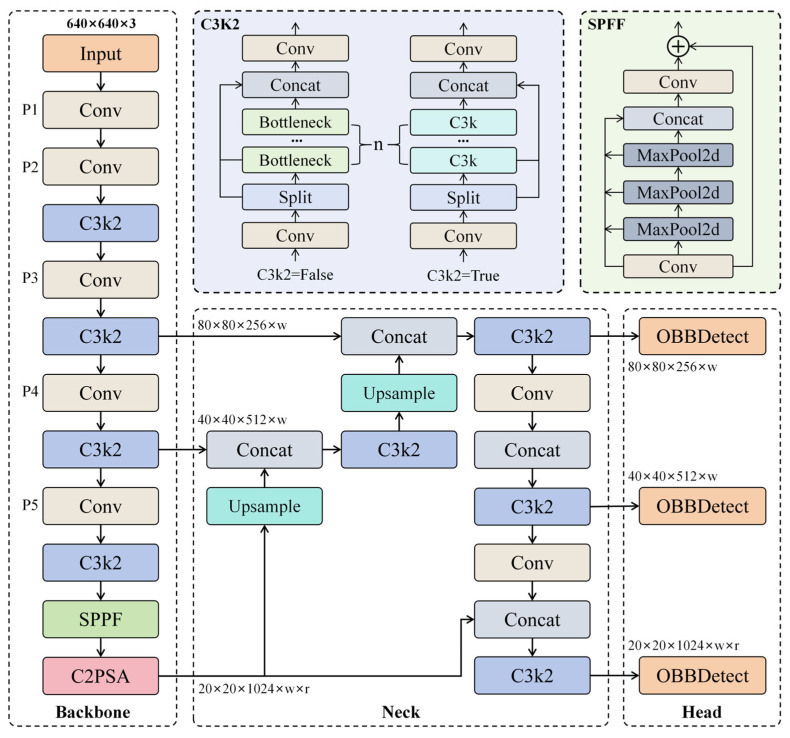
YOLO26-OBB network structure diagram.

This study formulated individual pig localization and feeding behavior recognition as an object detection task. YOLO26-OBB was used as the base model for subsequent visual feeding-intensity extraction. As shown in Figure 2, YOLO26-OBB adopts a single-stage detection architecture composed of a backbone, neck, and detection head. The backbone extracts multi-scale visual features. The C3k2, SPPF, and C2PSA modules enhance feature representation, enlarge the receptive field, and strengthen responses in informative regions. The neck integrates high-level semantic features and low-level localization features through upsampling, feature concatenation, and convolutional downsampling. This design improves the model’s ability to identify dense, occluded, and multi-pose pig targets. The detection head outputs oriented bounding boxes at three scales, namely 80 × 80, 40 × 40, and 20 × 20. Each box is represented as (x,y,w,h,θ,c,s), where θ describes the direction of the pig body’s long axis. Compared with HBB, OBB can better fit individual boundaries under different orientations. It may also reduce box overlap between adjacent pigs and background interference. Therefore, OBB is more suitable for frame-level feeding-state determination, feeding pig counting, and group feeding-intensity construction.

During model training, the input image size was set to 640 × 640. The number of training epochs was 300, the batch size was 16, and the initial learning rate was 0.01. All experiments were conducted on an NVIDIA GeForce RTX 4090 GPU. The software environment included Python 3.11, PyTorch 2.3.0, and CUDA 12.1.

### 2.4. Construction of Visual Feeding Intensity and Alignment with Feed Intake

To convert frame-level feeding recognition results into a continuous group-level behavioral indicator, the number of pigs identified as feeding in frame t is defined as the frame-level visual feeding count:

(1) $$C(t)=\sum_{i=1}^{n_{t}}I(y_{i}(t)=Feeding)$$

where $y_{i}(t)$ denotes the class label of the i-th detected object in frame t, and I(⋅) is the indicator function. The frame-level visual feeding counts were then aggregated within fixed time windows to obtain window-level visual feeding intensity:

(2) $$VI_{w}=\frac{1}{N_{w}}\sum_{t\in w}C(t)$$

where $VI_{w}$ denotes the visual feeding intensity within time window w, and $N_{w}$ is the number of valid video frames in that window. In this study, visual feeding intensity represents the number of pigs simultaneously feeding, as extracted by the vision model. To examine the relationship between the visual indicator and actual feed intake at different temporal scales, aggregation windows of 5, 10, 15, 20, 25, and 30 min were used. The visual feeding intensity within each window was then aligned with the corresponding actual feed intake increment.

### 2.5. Feeding Strategy Design

In this study, feeding strategy evaluation refers to an offline replay analysis rather than a real-time closed-loop feeding experiment. The analysis compared the temporal match between candidate allocation rules and video-derived feeding demand under a fixed total daily feed allocation. Only the allocation ratios among feeding periods were adjusted, while the total daily feed amount and the maximum feed share per period were kept unchanged.

A feeding event recorded by the feeder was defined as one feeding period. Each feeding period usually contained multiple video time windows. The video time windows were used to count the number of pigs feeding simultaneously from visual model outputs. Feeding periods were used as the basic units for feeding strategy generation and temporal matching evaluation. For reproducibility, the definitions, units, and sources of the variables and preset parameters used in the offline feeding-schedule replay are summarized in Appendix A, Table A1.

Let $B_{d}$ denote the total feed allocation on day d. Let $K_{d}$ denote the number of available feeding periods. Let $A_{d,k}$ denote the planned feed allocation in the k-th feeding period. All strategies satisfied the constant daily total allocation and per-period upper-limit constraints:

(3) $$\sum_{k=1}^{K_{d}}A_{d,k}=B_{d},\;0\leq A_{d,k}\leq \rho B_{d}$$

where ρ is the maximum feeding proportion for a single feeding period.

To obtain a behavioral demand signal at the feeding-period scale, the mean and peak numbers of simultaneously feeding pigs were weighted. Video-derived feeding demand is defined as:

(4) $$V_{d,k}=\omega C_{d,k}+(1-\omega )C_{d,k}^{\max}$$

where $C_{d,k}$ and $C_{d,k}^{max}$ denote the mean and peak numbers of simultaneously feeding pigs in the *k*-th feeding period on day *d*, respectively. In this formula, the mean and peak counts were selected to reflect complementary aspects of group feeding activity: the mean count represents sustained participation during a feeding period, whereas the peak count captures short high-demand bursts that may be diluted by period averaging. The parameter ω denotes the weight assigned to the mean count. The default value of ω was set to 0.40 in the main replay analysis, and its influence on the evaluation results was examined by varying ω from 0 to 1 in the sensitivity analysis. This indicator converts window-level video recognition outputs into video-derived feeding demand at the feeding-period scale and provides a unified behavioral reference for subsequent offline feeding-schedule evaluation.

To analyze the contribution of different information sources to feeding strategy design, strategy inputs were divided into four categories. Their definitions are provided in Table 1.

For strategies using F, VH, and VL, each information source was first normalized. The normalized sources were then combined by weights to obtain a strategy score. Let M⊆{F,VH,VL} denote any combination of information sources. The strategy score is calculated as:

(5) $$S_{d,k}^{M}=\sum_{m\in M}w_{m}N(X_{d,k}^{m}),\;\sum_{m\in M}w_{m}=1$$

where $X_{d,k}^{m}$ is the value of information source m in the k-th feeding period on day d. N(⋅) denotes normalization, and $w_{m}$ denotes the weight assigned to source m.

VE was used as current-period video feedback to generate the online budget allocation. Let $Q_{d,k}$ denote the early demand score obtained by normalizing VE. Let $R_{d,k}$ denote the remaining feed allocation before the start of the k-th feeding period. Let $L_{d,k}$ denote the number of remaining feeding periods. The online budget allocation corresponding to VE is defined as:

(6) $$A_{d,k}^{VE}=C_{\rho ,B_{d}}\left(\frac{R_{d,k}}{L_{d,k}}\left[1+\gamma (2Q_{d,k}-1)\right]\right)$$

where γ is the online feedback gain. This parameter controls the influence of video feedback on budget release intensity in the current feeding period. $C_{\rho ,B_{d}}(\cdot )$ denotes allocation correction under the constraints of constant daily total allocation and maximum single-period feeding proportion.

When VE is combined with F, VH, and VL, the final feed allocation is calculated as:

(7) $$A_{d,k}^{hybrid}=(1-\lambda )A_{d,k}^{M}+\lambda A_{d,k}^{VE}$$

where λ is the early-feedback weight. When only VE was used, the final feed allocation was $A_{d,k}^{VE}$.

Based on this framework, four representative feeding strategies were compared: a fixed equal-allocation strategy, a historical feed intake prior strategy (F), a video-aware causal strategy (F + VH + VL), and a video-aware hybrid strategy (F + VH + VL + VE). The fixed equal-allocation strategy used no historical or video information. The historical feed intake prior strategy reflected only long-term feeding patterns. The video-aware causal strategy incorporated historical video rhythms and same-day video feedback. This strategy satisfied the causal constraint of no future information leakage. The video-aware hybrid strategy further used current-period video feedback. This strategy was included to evaluate the gain from online behavioral signals in feeding-period allocation.

In the main replay analysis, the default parameters were ω= 0.40, ρ = 0.25, λ = 0.55, and γ = 0.90. These values were treated as preset engineering parameters rather than learned model parameters. Their influence on the evaluation metrics was examined in the parameter sensitivity analysis.

### 2.6. Evaluation Metrics and Statistical Analysis

The detection performance of the visual perception models was evaluated using precision, recall, mAP50, and mAP50-95. Model deployment efficiency was assessed using the number of parameters and the average inference time per image. Precision and recall are defined as follows:

(8) $$Precision=\frac{TP}{TP+FP}$$

(9) $$Recall=\frac{TP}{TP+FN}$$

The relationship between visual feeding intensity and actual feed intake was evaluated using Pearson’s correlation coefficient. To assess the effects of temporal aggregation scale and time lag, correlation coefficients were calculated under different time windows and lag conditions.

Leave-one-day-out cross-validation was further used to evaluate the predictive ability of visual feeding intensity for feed intake increments. In each iteration, data from one day were used as the test set. Data from the remaining days were used as the training set. Prediction performance is evaluated using $R^{2}$ and mean absolute error (MAE):

(10) $$R^{2}=1-\frac{\sum_{i=1}^{n}(y_{i}-{\hat{y}}_{i}{)}^{2}}{\sum_{i=1}^{n}(y_{i}-\bar{y}{)}^{2}}$$

(11) $$MAE=\frac{1}{n}\sum_{i=1}^{n}|y_{i}-{\hat{y}}_{i}|$$

where $y_{i}$ denotes the observed feed intake increment, and ${\hat{y}}_{i}$ denotes the predicted feed intake increment.

The temporal matching performance of different feeding strategies was evaluated using peak time difference, synchrony correlation, and mismatch rate. The demand peak refers to the feeding period in which pigs showed the strongest group-level feeding demand within a day, as indicated by the highest video-derived feeding-demand score $V_{d,k}$. The planned feeding peak refers to the feeding period receiving the largest scheduled feed allocation $A_{d,k}$. Peak time difference was calculated as the absolute difference between the start times of these two periods. Synchrony correlation was defined as the Pearson correlation coefficient between the scheduled allocation sequence and the video-derived feeding-demand sequence. Mismatch rate was defined as the proportion of periods with high planned feed allocation but low video-derived feeding demand, or high video-derived feeding demand but insufficient planned feed allocation. Together, these metrics evaluated the scheduling strategies in terms of peak timing, overall trend synchronization, and demand mismatch control.

## 3. Results

### 3.1. Performance of Feeding Behavior Recognition

As shown in Table 2, all models achieved high performance in the all-class detection task, although the results varied by bounding-box type and model architecture. Compared with Faster-RCNN and DETR-R50, the YOLO models generally had fewer parameters and shorter inference times. Within the same YOLO architecture, each OBB model achieved a higher mAP50-95 than its corresponding HBB model. For YOLOv8, YOLOv11, and YOLO26, mAP50-95 increased from 0.832, 0.829, and 0.830 to 0.903, 0.895, and 0.908, respectively. In contrast, differences in mAP50 were small, with all values remaining above 0.979. The improvement associated with OBB was therefore mainly reflected in localization performance under stricter IoU thresholds. Among all models, YOLO26-OBB achieved the highest mAP50-95 (0.908) and the shortest inference time (6.77 ms), with 9.75 M parameters, indicating a favorable overall balance between detection accuracy and inference efficiency.

For feeding and non-feeding recognition, all three OBB models maintained high detection performance (Table 3). In the feeding class, YOLO26-OBB achieved the highest precision (0.963), and its mAP50-95 was 0.919, equal to that of YOLOv8-OBB. In the non-feeding class, YOLO26-OBB achieved the highest mAP50-95 (0.897). Although YOLO26-OBB did not achieve the highest recall or mAP50 in every class, it maintained high mAP50-95 values for both behavior classes. It was therefore selected as the base model for subsequent extraction of visual feeding intensity. The recall of the feeding class was lower than that of the non-feeding class for YOLO26-OBB (0.926 vs. 0.963), probably because feeding behavior near the trough was more visually ambiguous. Feeding pigs often had partially occluded heads, body contact with neighboring pigs, or postures similar to pigs standing near the feeder, which could increase false negatives when actual feeding pigs were detected but classified as non-feeding.

**Figure 3:**
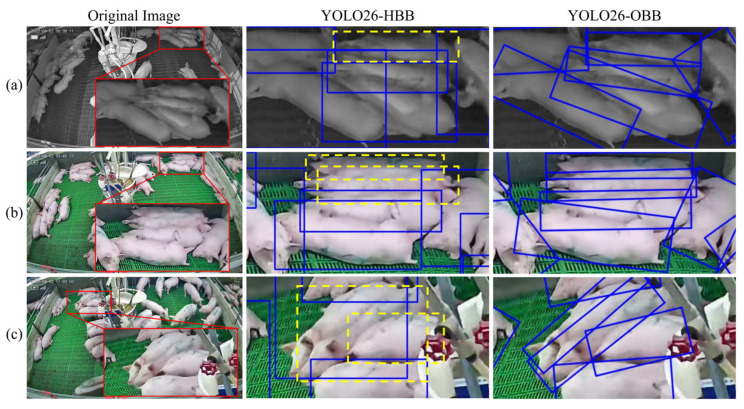
Comparison of detection results between YOLO26-HBB and YOLO26-OBB in densely clustered and partially occluded scenes. Panel (**a**) shows a representative nighttime scene, while panels (**b**,**c**) show representative daytime scenes. Red boxes indicate enlarged regions of the original images, blue boxes indicate pigs detected by the model, and yellow dashed boxes indicate pigs missed by YOLO26-HBB but detected by YOLO26-OBB.

The qualitative detection results further showed the advantage of OBB in densely clustered scenes. As shown in Figure 3, YOLO26-HBB failed to detect some pigs individually when animals were in contact, partially occluded, or oriented in different directions. The yellow dashed boxes indicate target regions missed by YOLO26-HBB. In the corresponding YOLO26-OBB results, these targets were detected with oriented bounding boxes. These results show that OBB better fitted pig body orientation and object boundaries, which helped reduce missed detections by HBB in densely clustered scenes.

**Figure 4:**
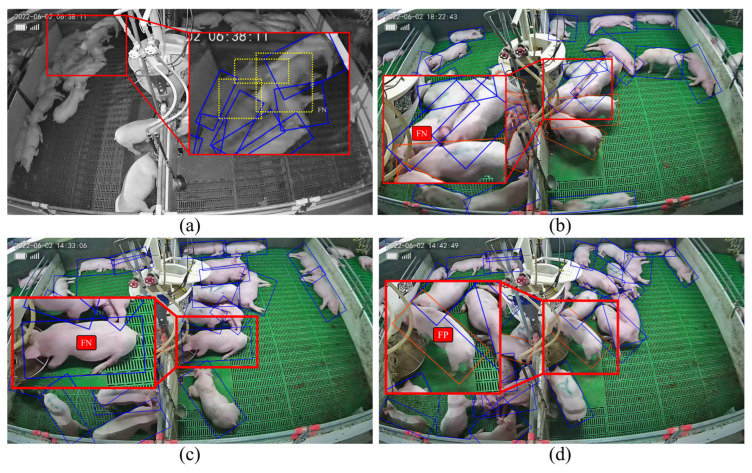
Typical detection errors and behavior recognition limitations of YOLO26-OBB in complex scenes: (**a**) missed detection; (**b**,**c**) false-negative (FN) cases; and (**d**) false-positive (FP) case. Red boxes indicate enlarged regions of the original images, yellow dashed boxes indicate missed pigs, orange-red bounding boxes indicate pigs detected by the model as feeding, and blue bounding boxes indicate pigs detected by the model as non-feeding.

YOLO26-OBB still showed limitations in complex farming scenes (Figure 4). Missed detections occurred under insufficient illumination and unclear object boundaries (Figure 4a). In behavior classification, some actual feeding pigs were classified as non-feeding pigs (Figure 4b,c), whereas some non-feeding pigs were classified as feeding pigs (Figure 4d). These errors indicate that end-to-end detection models remain affected by illumination, occlusion, blurred object boundaries, and similar behavioral postures when distinguishing feeding from non-feeding states. These examples explain why feeding recall was more affected than non-feeding recall, because missed feeding labels mainly occurred when head engagement with the trough was partially hidden or visually similar to non-feeding proximity to the feeder.

### 3.2. Association Between Visual Feeding Intensity and Feed Intake

Based on the feeding and non-feeding classification outputs from YOLO26-OBB, the number of pigs in the feeding state was counted in each frame. These frame-level counts were then aggregated within fixed time windows to construct a group-level visual feeding intensity sequence. In this study, visual feeding intensity was defined as the number of pigs simultaneously feeding, as extracted by the vision model. This indicator converted frame-level behavior recognition outputs into a continuous within-day curve of feeding activity, providing a basis for examining its association with actual feed intake.

**Figure 5:**
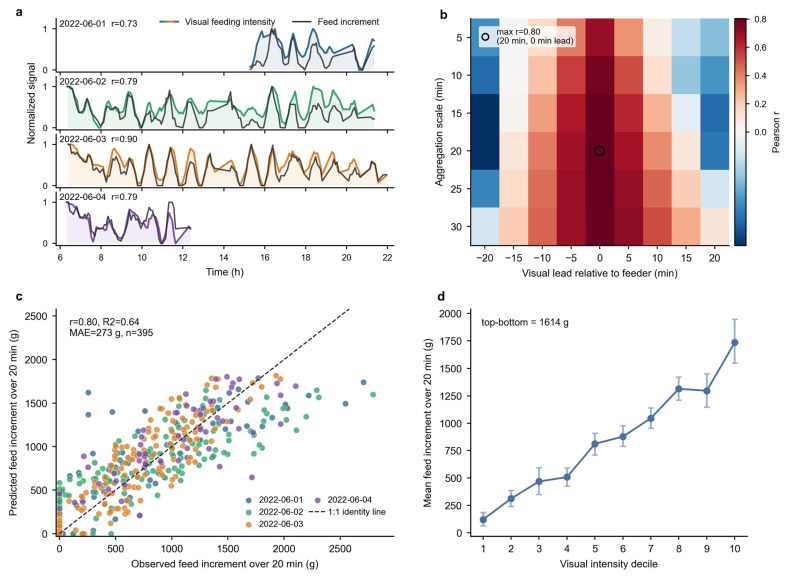
Multi-scale synchronization and quantitative association between visual feeding intensity and actual feed intake increments. (**a**) Within-day time series of normalized visual feeding intensity and feed intake increments across different dates. Visual feeding intensity represents the number of pigs simultaneously feeding, as extracted by the vision model. (**b**) Pearson correlation coefficients between visual feeding intensity and feed intake increments under different temporal aggregation scales and time-lag conditions. (**c**) Leave-one-day-out cross-validation prediction results based on visual feeding indicators. (**d**) Mean feed intake increment response after grouping visual feeding intensity into deciles.

As shown in Figure 5a, normalized visual feeding intensity and feed intake increments showed similar within-day fluctuation patterns across dates, with peaks and troughs generally synchronized in most time periods. Pearson correlation coefficients within individual dates ranged from 0.73 to 0.90. Lag-scale correlation analysis further showed that the association between visual feeding intensity and feed intake increments depended on the temporal aggregation scale. Among the tested aggregation windows, the 20 min window produced the strongest zero-lag Pearson correlation between visual feeding intensity and feed-intake increment (*r* = 0.804; Figure 5b). Correlations decreased when positive or negative time lags were introduced, indicating that the two data streams were most closely aligned within the same 20 min interval.

To evaluate whether the visual indicator could characterize changes in feed intake, leave-one-day-out cross-validation was performed using visual feeding intensity and its temporally aggregated features as predictors. RidgeCV ridge regression was used as the prediction model. Predicted 20 min feed intake increments were correlated with the observed increments in the pooled leave-one-day-out evaluation (*r* = 0.80, *R*^2^ = 0.64, MAE = 273 g, *n* = 395; Figure 5c). Day-level prediction performance is summarized in Table 4. At the day level, leave-one-day-out prediction performance remained positive across all observation days, with r ranging from 0.749 to 0.890, *R*^2^ from 0.559 to 0.742, and MAE from 192.7 to 357.6 g. These results indicate that the association between visual feeding intensity and feed-intake increments was not driven by a single observation day, although the number of observation days remained limited.

After visual feeding intensity was divided into decile groups, the mean feed intake increment increased gradually with visual intensity level. The difference between the highest- and lowest-intensity groups was 1614 g (Figure 5d). These results indicate that the number of pigs simultaneously feeding, as extracted by the vision model, captured within-day variation in group feed intake.

Together, these analyses support the use of visual feeding intensity as an indicator of within-day changes in group feed intake. This indicator was therefore aggregated into video-derived feeding demand at the feeding-period scale for subsequent evaluation of feeding scheduling strategies.

### 3.3. Matching Evaluation of Feeding Strategies Based on Video-Derived Feeding Demand

Based on the synchronized relationship between visual feeding intensity and actual feed intake increments, visual feeding intensity was further aggregated at the feeding-period scale to construct a video-derived feeding-demand indicator. This indicator was used as a group-level proxy for feeding demand in an offline replay analysis of temporal matching between candidate allocation rules and video-derived demand.

#### 3.3.1. Comparison of Temporal Matching Performance Among Feeding Strategies

**Figure 6:**
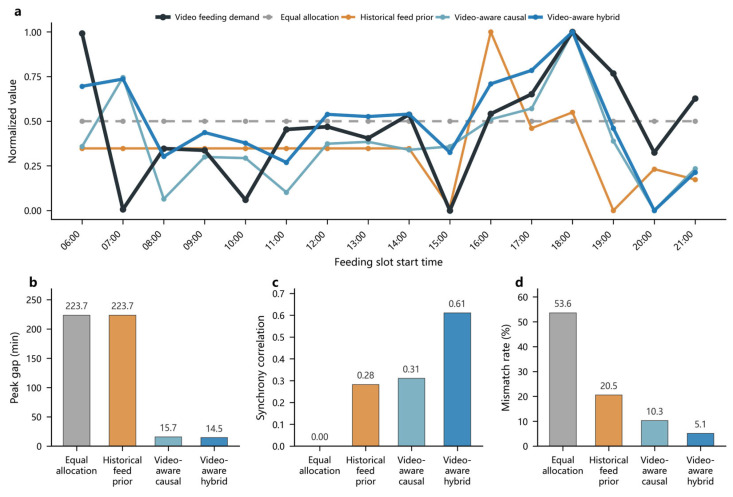
Comparison of within-day alignment between different feeding-period scheduling strategies and video-derived feeding demand. (**a**) Normalized time-series curves of typical within-day video-derived feeding demand and different scheduling strategies. (**b**) Peak time difference in different strategies. (**c**) Synchronization correlation between different strategies and video-derived feeding demand. (**d**) Mismatch rate of different strategies.

As shown in Figure 6, the fixed equal-allocation strategy remained constant across feeding periods and did not respond to intraday fluctuations in video-derived feeding demand. It showed a peak time difference of 223.69 min, a synchronization correlation of 0.000, and a mismatch rate of 53.57%. When only the historical feed-intake prior was used, the mismatch rate decreased to 20.54%, and the synchronization correlation increased to 0.282, but the peak time difference remained 223.67 min. These results indicate that historical feeding patterns improved the overall allocation structure, but did not capture same-day temporal variation in the peak of video-derived feeding demand.

After video-based behavioral signals were introduced, temporal consistency between feeding strategies and video-derived feeding demand improved. The video-aware causal strategy reduced the peak time difference to 15.70 min, lowered the mismatch rate to 10.27%, and increased the synchronization correlation to 0.311. After early video feedback from the current feeding period was added, the video-aware hybrid strategy showed the best overall performance. Its peak time difference was 14.51 min, synchronization correlation was 0.611, and mismatch rate was 5.13%. Compared with the historical feed-intake prior strategy, the hybrid strategy reduced the peak time difference by 209.16 min and the mismatch rate by 15.40 percentage points. These results indicate that video feedback provided additional behavioral information for feeding-period allocation beyond historical feeding patterns.

#### 3.3.2. Ablation Analysis of Feedback Information Sources

To clarify the contribution of different information sources, a combinatorial ablation analysis was performed for F, VH, VL, and VE (Table 5). In the single-factor results, F mainly reduced the mismatch rate but showed limited improvement in peak localization. VH and VL reduced the peak time difference to 43.49 min and 45.89 min, respectively, although their synchronization correlations and mismatch control remained limited. VE achieved the highest synchronization correlation among the single-factor inputs (0.656) and reduced the mismatch rate to 0.00%, but its peak time difference remained large (253.90 min). This pattern occurred because mismatch rate and peak time difference evaluate different aspects of temporal matching. Under the quartile-based mismatch definition, VE reduced high–low allocation mismatches, but the largest allocation generated by VE alone did not coincide with the daily demand peak. These results indicate that early video feedback helped reduce period-level mismatch, but it was insufficient to localize the daily demand peak when used without historical or lagged demand information.

The combinatorial ablation results further showed that video-related information was the main source of improved scheduling alignment. VH + VL reduced the peak time difference to 15.70 min and the mismatch rate to 10.27%, indicating that historical video rhythms and same-day lagged video feedback contributed to demand-peak localization. By contrast, F + VH had a mismatch rate of 27.68%, which was not lower than that obtained using the historical feed intake prior alone. Thus, simply combining historical feed intake patterns with historical video rhythms did not necessarily provide additional gain. In some combinations, long-term feed intake priors appeared to weaken the contribution of same-day video feedback. Overall, VH, VL, and VE contributed more clearly to alignment between scheduling strategies and video-derived feeding demand.

#### 3.3.3. Sensitivity Analysis of Key Parameters

To evaluate the dependence of the video-aware hybrid strategy on parameter settings, a full-factorial sensitivity analysis was performed. Four parameters were examined: the mean-weight parameter for video-derived feeding demand, *ω*; the maximum single-period feeding proportion, *ρ*; the early-feedback weight, *λ*; and the online feedback gain, *γ*. The parameter settings are shown in Table 6. For each parameter combination, video-derived feeding demand was recalculated and the video-aware hybrid strategy was rerun, while the constraints on constant daily total allocation and maximum single-period allocation were kept unchanged.

The results showed that peak localization and trend synchronization were relatively sensitive to parameter changes, whereas mismatch control remained more stable (Table 7). Across parameter combinations, the peak time difference ranged from 14.51 to 255.09 min, and the synchronization correlation ranged from 0.223 to 0.782. By contrast, the mismatch rate ranged from 0 to 13.84%, with a median of 5.13%. These results suggest that parameter settings mainly affected the ability of the strategy to capture feeding peaks and intraday trends, while feed allocation–demand mismatch control was maintained across a relatively broad parameter range.

#### 3.3.4. Robustness Analysis Under Perturbations in Visual Counts

To evaluate the effect of visual counting errors on feeding strategy evaluation, multiplicative random perturbations of 0%, 5%, 10%, 20%, and 30% were applied to early visual counts, visual mean counts, and visual maximum counts. Except for the 0% perturbation, each noise level was repeated 100 times. The video-aware hybrid strategy was then rerun while keeping the total daily feed allocation and maximum single-period allocation unchanged.

**Figure 7:**
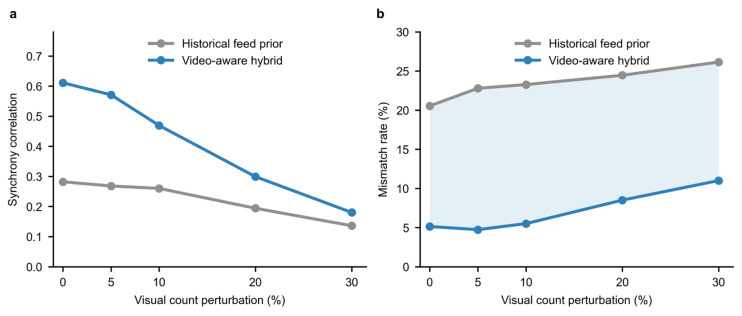
Robustness of the video-aware hybrid strategy under visual count perturbations. (**a**) Changes in synchronization correlation across visual count perturbation levels; (**b**) changes in mismatch rate across visual count perturbation levels. In (**b**), the light-blue shaded area denotes the reduction in mismatch rate achieved by the video-aware hybrid strategy relative to the historical feed prior at the same perturbation level, highlighting its robustness advantage under visual count noise.

The perturbation experiment showed that visual counting errors reduced synchronization between the hybrid strategy and trends in video-derived feeding demand (Figure 7). As the perturbation level increased from 0% to 30%, the synchronization correlation of the hybrid scheduling strategy decreased from 0.611 to 0.180. For mismatch control, however, the hybrid strategy still outperformed the historical feed intake prior at all noise levels. Under 20% perturbation, the mismatch rate of the hybrid strategy was 8.50%, lower than the 24.46% obtained with the historical feed intake prior. Under 30% perturbation, the mismatch rate of the hybrid strategy was 11.00%, still lower than the 26.15% obtained with the historical feed intake prior. These results suggest that visual counting errors mainly affected trend synchronization accuracy, but did not eliminate the advantage of video-based behavioral signals in reducing feed allocation–demand mismatch.

Overall, video-derived feeding demand can serve as a behavioral feedback reference at the feeding-period scale and can be used to improve the temporal match between planned feed allocation and intraday group feeding activity. The ablation, parameter sensitivity, and noise perturbation analyses provided consistent evidence from different perspectives. Video-based behavioral signals improved the evaluation performance of the main feeding strategy and retained an advantage in mismatch control under parameter variation and visual counting errors.

## 4. Discussion

### 4.1. Adaptability of Oriented Bounding Boxes to Group Feeding Scenarios

This study suggests that formulating pig localization and feeding/non-feeding recognition as an oriented object detection task is well suited to group feeding scenarios with dense distributions, partial occlusion, and variation in posture. Near feeding troughs, pigs are often arranged in multiple directions. Horizontal bounding boxes may include background regions and adjacent individuals, increasing the risk of missed detections or class confusion [19,22]. Complex pen environments may also reduce the stability of pig location, posture, and feeding behavior recognition [24,29]. By contrast, oriented bounding boxes can better align with the longitudinal body axis of pigs, reducing invalid regions and interference from neighboring targets. This provides more reliable individual boundaries for frame-level feeding counts.

Recent studies on arbitrary-oriented object detection have shown that orientation representation, classification-based angle modeling, and prediction decoupling can improve localization stability for multi-directional targets [37,38]. Studies of feeding behavior recognition, posture recognition, and video-based behavior recognition in commercial pig houses also suggest that deep learning models are gradually moving from offline recognition toward use in complex farming scenarios [39,40,41]. These results support the feasibility of OBB-based feeding/non-feeding recognition in the annotated dense-pen images used in this study. Their generalization across farms, camera views, pig ages, lighting conditions, and feeding systems still requires further validation.

### 4.2. Behavioral Meaning of Visual Feeding Intensity

Visual feeding intensity may characterize within-day dynamics in group feed intake because the number of pigs feeding simultaneously represents group feeding participation within a given time window. Short-term group feed intake is jointly shaped by the number of pigs at the trough, feeding duration, feeding rhythm, and individual differences [11,12]. Studies of feeding behavior indices and intake structure also suggest that feeding frequency, feeding duration, and intake distribution can describe feeding behavior in finishing pigs [13,14]. Aggregating frame-level feeding recognition results into window-level visual feeding intensity therefore converts discrete behavioral classifications into a continuous curve of group feeding activity, which can be temporally aligned with feed intake increments recorded by the feeder.

In this study, visual feeding intensity showed clear synchronization with actual feed intake increments in within-day trends. This finding suggests that video-captured group feeding activity reflected concurrent changes in feed consumption. This relationship supports the use of visual feeding intensity as a group-level measure of immediate feeding activity and as a proxy for short-term feeding demand. However, it should not be interpreted as a deterministic predictor of individual intake, future intake, or demand responses after a changed feeding schedule is implemented. Its main value lies in providing continuous, low-intrusion behavioral feedback for offline feeding-schedule evaluation and future schedule design. This interpretation is consistent with previous work showing that feeding patterns can support welfare assessment, feed-efficiency prediction, and production-status characterization [15,16]. Recent studies have also used video data and automatic behavior recognition to describe behavioral sequences in finishing pigs, while CNN-LSTM-based behavior classification in group-housed pigs suggests that temporal features are important for interpreting changes in group behavior [42,43]. RFID-based online feeding monitoring can provide fine-grained individual feeding data [18], whereas the video-derived indicator used here provides a complementary group-level behavioral signal without requiring individual electronic identification.

### 4.3. Engineering Significance of Video-Derived Feeding Demand for Feeding Strategy Optimization

Aggregating visual feeding intensity into video-derived feeding demand at the feeding-period scale may extend video monitoring from behavioral observation to a feedback reference for feeding strategies. Its engineering value lies in compressing high-frequency, continuous recognition outputs into period-level demand signals that can be combined with feeding periods, single-period allocation limits, and daily total allocation constraints. The value of precision pig farming technologies lies not only in data collection, but also in converting animal-state information into process-level information for management decisions [1,3]. Precision feeding has similarly been used to estimate nutrient requirements in real time and optimize diet supply [6,7]. From the perspective of period-level demand matching, this study established a conversion pathway from video recognition outputs to period-level feeding demand and feeding strategy evaluation. This pathway broadens the application of video-based behavioral signals in feeding management.

In production practice, feeding strategy design involves more than determining the daily feed allocation. It also requires improving the temporal match between feeding periods and group feeding activity [8,9,10]. Fixed equal allocation is difficult to adapt to intraday fluctuations. Historical feed intake priors can reflect long-term patterns, but their adaptability to daily environmental conditions, herd status, and short-term behavioral rhythms is limited. By contrast, video-aware strategies incorporate same-day behavioral signals. Under a constant daily allocation, these strategies improved synchronization between planned feed allocation and video-derived feeding demand. This finding suggests that video-based behavioral signals can supplement fixed or experience-based feeding with behavioral feedback and may provide a basis for developing online closed-loop feeding systems. However, the present analysis evaluated temporal matching in an offline replay setting. Whether such allocation rules improve residual feed control, feed-use efficiency, growth performance, or welfare after implementation remains to be tested in a real closed-loop feeding system.

### 4.4. Limitations and Future Research Directions

This study has several limitations. First, the feeding-demand and strategy analyses were based on one pen, one batch, pigs aged 59–62 days, and 72 h of video. This design enabled a controlled proof-of-concept evaluation of the proposed workflow. However, the dataset covered limited variation in pen layout, camera geometry, feeding equipment, lighting conditions, season, and management events. Further validation across multiple pens, farms, growth stages, batches, seasons, and environmental conditions is therefore needed. For practical deployment, scenario adaptability, equipment availability, management workflow, and deployment cost should also be considered [4,5]. In addition, synchronized ambient-temperature and humidity records were not available in the current dataset. Therefore, we could not evaluate whether the video-demand relationship or scheduling performance changed under different thermal conditions.

The annotated dataset was also limited in size and class distribution. The model may still produce missed detections or misclassifications under low light, severe occlusion, blurred boundaries, and similar postures. This is consistent with previous video-based pig behavior recognition studies, which reported that complex pen environments can affect model stability and generalization [20,23].

Another limitation is that the image annotations were produced by one trained annotator and manually reviewed according to predefined feeding/non-feeding criteria. Although feeder-timing records were not used during image labeling, feeding status in dense trough scenes can still be ambiguous when the head is occluded or when pigs remain close to the trough without clear feeding engagement. Therefore, inter-annotator agreement could not be calculated in the present dataset. Future studies should include independent re-annotation of a representative image subset and report agreement statistics, such as Cohen’s kappa for feeding/non-feeding classification and IoU-based agreement for oriented bounding boxes.

In addition, feeding-schedule evaluation in this study was based on offline replay rather than real implementation. The effects of a behavior-aware schedule on future pig behavior, residual feed, feed-use efficiency, production performance, and economic return have not yet been validated. Such validation requires a closed-loop feeding experiment in which the allocation rule is implemented and its feedback effects are measured over time [7,10].

Future studies should validate the stability of visual feeding intensity across multiple farms, age groups, and longer observation periods. Model robustness may be improved by integrating temporal modeling, object tracking, multi-camera fusion, and domain adaptation. Previous studies have shown that computer vision and localization systems can support continuous monitoring of group-housed pigs, although long-term tracking may still be affected by trajectory interruptions, environmental changes, and equipment deployment conditions [44,45]. Future work could further integrate feeder records, environmental sensors, individual identification, markerless pose estimation, and three-dimensional vision [30,31,32]. Such integration would support the development of an online feeding scheduling system with real-time perception, dynamic allocation, and safety constraints. Its application value should then be validated under practical production conditions.

## 5. Conclusions

This study developed a framework for sensing feeding demand in pig groups and evaluating feeding strategies using rotated object detection. The framework enabled a stepwise conversion from frame-level feeding-state recognition to window-level visual feeding intensity and feeding-period-scale video-derived feeding demand. In dense feeding scenarios, YOLO26-OBB achieved a favorable balance between detection accuracy and inference efficiency, with an mAP50-95 of 0.908 and an inference time of 6.77 ms per image. Visual feeding intensity derived from this model remained strongly synchronized with actual feed intake increments, with a correlation coefficient of 0.80 under a 20 min aggregation window and no time lag. The video-aware hybrid strategy further improved the temporal match between planned feed allocation and video-derived feeding demand, reducing the peak time difference to 14.51 min, increasing the synchronization correlation to 0.611, and reducing the mismatch rate to 5.13%. Overall, the results indicate that video-based behavioral signals can serve as a group-level proxy for feeding activity and can support offline evaluation of temporal matching between planned allocation and video-derived demand. These findings provide a methodological basis for behavior-aware feeding-schedule design, but they do not yet validate practical deployment. Future studies should test the robustness and production effects of this approach over longer periods, multiple pens, different batches and seasons, and real closed-loop feeding systems.

## Figures and Tables

**Table 1:** Definitions of information sources used as inputs for feeding strategies.

Symbol	Input Source	Definition
F	Historical intake prior	Mean feed intake proportion for the same feeding period on historical days
VH	Historical video prior	Video-derived feeding demand for the same feeding period on historical days
VL	Lagged video feedback	Video-derived feeding demand in the previous feeding period on the same day
VE	Current-period video feedback	Mean feeding-pig count in the first two valid windows of the current period

**Table 2:** Performance comparison of different HBB and OBB detection models.

Models	Type	P	R	mAP50	mAP50-95	Inference Time	Param
Faster-RCNN	All	0.958	0.942	0.979	0.748	15.90 ms	41.35 M
DETR-R50	All	0.959	0.954	0.983	0.798	18.52 ms	41.56 M
YOLOv8-HBB	All	0.972	0.959	0.985	0.832	8.41 ms	11.13 M
YOLOv11-HBB	All	0.954	0.969	0.983	0.829	7.51 ms	9.41 M
YOLO26-HBB	All	0.944	0.962	0.98	0.830	7.93 ms	9.47 M
YOLOv8-OBB	All	0.96	0.961	0.985	0.903	9.08 ms	11.41 M
YOLOv11-OBB	All	0.953	0.961	0.986	0.895	8.97 ms	9.70 M
YOLO26-OBB	All	0.961	0.945	0.984	0.908	6.77 ms	9.75 M

**Table 3:** Comparison of recognition performance among OBB detection models for feeding and non-feeding classes.

Models	Type	P	R	mAP50	mAP50-95
YOLOv8-OBB	Feeding	0.948	0.944	0.981	0.919
YOLOv8-OBB	Non-Feeding	0.971	0.978	0.99	0.887
YOLOv11-OBB	Feeding	0.951	0.935	0.984	0.913
YOLOv11-OBB	Non-Feeding	0.954	0.986	0.987	0.878
YOLO26-OBB	Feeding	0.963	0.926	0.98	0.919
YOLO26-OBB	Non-Feeding	0.958	0.963	0.988	0.897

**Table 4:** Day-level leave-one-day-out prediction performance.

Date	*n* Windows	*r*	*R* ^2^	MAE (g)
1 June 2022	52	0.749	0.559	357.6
2 June 2022	140	0.812	0.609	332.4
3 June 2022	144	0.890	0.742	192.7
4 June 2022	59	0.781	0.603	253.9
Pooled	395	0.802	0.643	273.1

**Table 5:** Ablation results for scheduling information sources.

Included Factors	Peak Time Difference (min)	Synchronization Correlation	Mismatch Rate (%)
None	223.69	0.000	53.57
F	223.67	0.282	20.54
VH	43.49	0.273	24.11
VL	45.89	0.142	13.39
VE	253.90	0.656	0.00
F + VH	73.87	0.241	27.68
F + VL	181.39	0.242	11.83
F + VE	194.69	0.609	1.56
VH + VL	15.70	0.277	10.27
VH + VE	14.51	0.635	1.56
VL + VE	29.00	0.536	5.13
F + VH + VL	15.70	0.311	10.27
F + VH + VE	44.88	0.600	1.56
F + VL + VE	180.20	0.578	1.56
VH + VL + VE	14.51	0.599	5.13
F + VH + VL + VE	14.51	0.611	5.13

**Table 6:** Parameter settings used in the sensitivity analysis of the video-aware hybrid strategy.

Parameter	Description	Values
*ω*	Mean-count weight in demand estimation	0, 0.25, 0.40, 0.50, 0.75, 1.00
*ρ*	Maximum feed share per slot	0.20, 0.25, 0.30, 0.35
*λ*	Weight of early video feedback	0, 0.25, 0.55, 0.75, 1.00
*γ*	Online feedback gain	0.60, 0.90, 1.20

**Table 7:** Parameter sensitivity analysis of the video-aware hybrid strategy.

Evaluation Metric	Minimum	Median	Maximum
Peak time difference (min)	14.51	59.29	255.09
Synchronization correlation	0.223	0.505	0.782
Mismatch rate (%)	0.00	5.13	13.84
Allocation MAE	0.011	0.022	0.037
Weighted video-demand index	0.473	0.533	0.616

## Data Availability

Because the raw data involve production privacy on commercial pig farms, the data presented in this study are available upon request from the corresponding author.

## Appendix A

**Table A1:** Variables and parameters used in the offline feeding-schedule replay.

Symbol	Meaning	Unit/Range	Source/Status
$B_{d}$	Total daily feed allocation on day *d*	g	Known input from feeder records
$K_{d}$	Number of available feeding periods on day *d*	count	Known input from feeder events
$A_{d,k}$	Feed allocated to feeding period *k* on day *d*	g	Calculated output
ρ	Maximum feed share allowed in one period	0–1	Preset constraint, default 0.25
$C_{d,k}$	Mean number of feeding pigs in period *k*	pigs	Calculated from video windows
$C_{d,k}^{max}$	Peak number of feeding pigs in period *k*	pigs	Calculated from video windows
ω	Weight assigned to the mean count in $V_{d,k}$	0–1	Preset parameter, default 0.40
$V_{d,k}$	Video-derived feeding demand	relative score	Calculated behavioral proxy
λ	Weight of early video feedback	0–1	Preset parameter, default 0.55
γ	Online feedback gain	positive scalar	Preset parameter, default 0.90
